# Preliminary results in the analysis of the immune response after aneurysmal subarachnoid hemorrhage

**DOI:** 10.1038/s41598-020-68861-y

**Published:** 2020-07-16

**Authors:** Jorge A. Roa, Deepon Sarkar, Mario Zanaty, Daizo Ishii, Yongjun Lu, Nitin J. Karandikar, David M. Hasan, Sterling B. Ortega, Edgar A. Samaniego

**Affiliations:** 1 Department of Neurology, University of Iowa Hospitals and Clinics, Iowa City, Iowa USA; 2 Department of Neurosurgery, University of Iowa Hospitals and Clinics, Iowa City, Iowa USA; 3 Department of Pathology, University of Iowa Hospitals and Clinics, Iowa City, Iowa USA; 4 Department of Radiology, University of Iowa Hospitals and Clinics, Iowa City, Iowa USA

**Keywords:** Adaptive immunity, Chemokines, Cytokines, Inflammation, Innate immune cells, Innate immunity, Immunology, Neurology, Neurological disorders

## Abstract

Cerebral vasospasm (VSP) is a common phenomenon after aneurysmal subarachnoid hemorrhage (aSAH) and contributes to neurocognitive decline. The natural history of the pro-inflammatory immune response after aSAH has not been prospectively studied in human cerebrospinal fluid (CSF). In this pilot study, we aimed to identify specific immune mediators of VSP after aSAH. Peripheral blood (PB) and CSF samples from patients with aSAH were prospectively collected at different time-points after hemorrhage: days 0–1 (acute); days 2–4 (pre-VSP); days 5–9 (VSP) and days 10 + (post-VSP peak). Presence and severity of VSP was assessed with computed tomography angiography/perfusion imaging and clinical examination. Cytokine and immune mediators’ levels were quantified using ELISA. Innate and adaptive immune cells were characterized by flow cytometry, and cell counts at different time-points were compared with ANOVA. Confocal immunostaining was used to determine the presence of specific immune cell populations detected in flow cytometry. Thirteen patients/aneurysms were included. Five (38.5%) patients developed VSP after a mean of 6.8 days from hemorrhage. Flow cytometry demonstrated decreased numbers of CD45+ cells during the acute phase in PB of aSAH patients compared with healthy controls. In CSF of VSP patients, NK cells (CD3-CD161 +) were increased during the acute phase and progressively declined, whereas CD8+CD161+ lymphocytes significantly increased at days 5–9. Microglia cells (CD45dimCD11b +) increased over time after SAH. This increase was particularly significant in patients with VSP. Levels of VEGF and MMP-9 were consistently higher in VSP patients, with the highest difference occurring at the acute phase. Confocal immunostaining demonstrated the presence of CD8+CD161+ lymphocytes in the arterial wall of two unruptured intracranial aneurysms. In this preliminary study, human CSF showed active presence of innate and adaptive immune cells after aSAH. CD8+CD161+ lymphocytes may have an important role in the inflammatory response after aneurysmal rupture and were identified in the aneurysmal wall of unruptured brain aneurysms. Microglia activation occurs 6 + days after aSAH.

## Introduction

Brain injury after aneurysmal subarachnoid hemorrhage (aSAH) is a multimodal process that includes early brain injury and delayed cerebral ischemia (DCI). The mechanisms that lead to DCI are not fully understood^1^. The pathogenesis of DCI is hypothesized to include cerebral vasospasm (VSP), cortical spreading ischemia, microthrombosis and constriction of the microcirculation^2^.

About two-thirds of patients with aSAH develop VSP 3–14 days after initial rupture^3^. Heterogeneous evidence has suggested several mechanisms as potential contributors of VSP including thickness, density, location and persistence of subarachnoid blood^4,5^. Red blood cells in the subarachnoid space are hemolyzed, releasing hemoglobin, bilirubin and oxygen free radicals. These toxic molecules induce increased expression of endothelin-1 and reduced levels of nitrous oxide, leading to endothelial cell dysfunction and VSP^6–8^. Although angiographic VSP eventually resolves, arterial fibrosis, endothelial thickening and reduced arterial compliance may persist over time and contribute to the poor clinical outcomes observed in patients with DCI^9^.

The inflammatory response following aSAH leads to increased permeability of the blood–brain barrier (BBB) and increased expression of cellular adhesion molecules. These molecules allow infiltration of peripheral leukocytes that phagocytose red blood cells and debris in the cerebrospinal fluid (CSF)^10–12^. Activated leukocytes secrete several cytokines that promote activation of central nervous system-resident macrophages (microglia) and trigger secondary injury of the brain parenchyma^13^.

Cells of the innate immune system such as neutrophils, monocytes and macrophages, have been found in high numbers and in highly activated states in post-hemorrhagic CSF^14^. Increased CSF concentrations of neutrophils, myeloperoxidase and NADPH oxidase have been documented in patients with VSP^15^. On the other hand, little is known about the role of the adaptive immunity in aSAH. Histological studies from clipped aneurysms’ specimens and autopsies have found lymphocyte infiltration in the aneurysmal wall^16,17^. However, T and B cells have only rarely been found in the CSF of aSAH patients^18,19^. In this study, we aimed to characterize the evolution of the innate and adaptive immune responses after aSAH, and determine if there is a correlation between immune cell activation and the development of VSP.

## Methods

### Patients and data collection

After approval by the University of Iowa Institutional Review Board, CSF and peripheral blood (PB) samples of patients with aSAH were prospectively collected. All experiments were performed in accordance with relevant guidelines and regulations. Inclusion criteria were as follows: (1) clinical presentation within 24 h after aSAH, (2) evidence of aneurysmal etiology of the hemorrhage documented in computed tomography angiography (CTA), magnetic resonance angiography or digital subtraction angiography, and (3) placement of an external ventricular drain (EVD) for acute management of post-hemorrhagic hydrocephalus. Subjects with history of congenital or acquired immunosuppression, central nervous system infection, presumed bacteremia, endocarditis, autoimmune diseases, systemic inflammatory diseases or current treatment with immunosuppressants or immunomodulators were excluded.

### CSF and PB sampling

Informed consent was obtained before sample collection. CSF samples were collected at four different time-points from the index hemorrhage (Supplemental Fig. 1): days 0–1 (acute phase); days 2–4 (pre-VSP); days 5–9 (VSP) and days 10 + (post-VSP peak). Five mL of CSF were slowly drawn from the EVD under continuous intracranial pressure monitoring and following standard aseptic techniques. The first 1.5 mL of CSF were discarded (dead space). A PB sample was collected simultaneously from the same patient at the first time-point, and PB samples from 6 control subjects without intracranial aneurysms were collected for comparison. The CSF was sampled until the EVD was discontinued (days 15–20 post-SAH).

### Leukocyte subsets and gating strategy

Automated CSF leukocyte counts were performed on ABX micros 60 (Horiba, Montpellier, France). Cells were washed twice with phosphate-buffered saline and immune-stained for 30 min at 4 °C with the following panel of antibodies: anti-CD45-BV786, anti-CD8-BV510, antiCD66b-BV421, anti-CD45-PerCp5.5 (BD Bioscience, San Jose, CA, USA); anti-CD3-FITC, anti-CD19-PECy7, anti-CD11b-PECy5, anti-CD161-PE, anti-CD14-Alexa700, anti-CD11c-APC (Tonbo Biosciences, San Diego, CA, USA). Optimal antibody concentrations were previously defined by titration^20^. The cells were washed and suspended in 1% paraformaldehyde. Flow cytometry data was collected on a BD FACS LSR flow cytometer equipped with four lasers. Analyses of leukocyte subsets on CSF and PB samples were conducted by 13 flow cytometry within 30 min from collection. BD FACS Diva software was used for data acquisition and Flow Jo v10 (Becton–Dickinson Bioscience, San Jose, CA, USA) was used for data analysis. The gating strategy is depicted in Supplemental Fig. 2.

### Enzyme linked immunoassay

CSF levels of several mediators and cytokines were quantified using Enzyme Linked Immunoassay (ELISA) arrays. This was done to better characterize the interactions and functional profile of immune cells after aSAH. Although several cytokines have been associated with occurrence of VSP, we analyzed the most often cited immune mediators as follows: vascular endothelial growth factor (VEGF), interleukin-6 (IL-6), tumor necrosis factor-alpha (TNF-α) (Tonbo Biosciences, San Diego, CA, USA), and matrix metalloproteinase-9 (MMP-9) (R&D Systems Minneapolis, MS, USA).

### Immunofluorescence imaging

Samples of 2 previously clipped unruptured intracranial aneurysms were processed. Aneurysm tissue was fixed in 10% Neutral-Buffered Formalin within one hour of surgical excision. Formalin-fixed, paraffin-embedded tissue specimens were prepared, stored at room temperature and sectioned in slices of 4–8 μm thickness. Slides were deparaffinized and subjected to citrate buffer antigen retrieval. Then, tissue sections were stained using primary and secondary antibodies anti-human CDs to determine the presence of immune cells identified in flow cytometry. Images were acquired using a Zeiss LSM 710 confocal microscope with Zen 2010 software (Zeiss Group, Oberkochen, Germany).

### Clinical outcome

VSP was defined as the development of a new focal neurological deficit that correlated with angiographic evidence of arterial narrowing on CTA/computed tomography perfusion imaging. Confounders of neurological deterioration such as hydrocephalus, fever, seizures and infection were ruled out. Based on previous literature and current guidelines^21,22^, degree of VSP was classified in three different categories using the following clinical and radiographic criteria: (1) mild =  ≤ 33% reduction in arterial diameter and self-limited neurological deficits; (2) moderate = 34% to 66% reduction in arterial diameter and clinical improvement with triple-H therapy; and (3) severe =  ≥ 67% reduction in arterial diameter requiring endovascular intra-arterial therapy. As standard of care, all patients were monitored in the neuro-intensive care unit and received nimodipine 60 mg every 4 h.

### Statistical analysis

Measures of central tendency and dispersion were calculated using descriptive statistics. Brown-Forsythe test was performed to assess differences in standard deviations and confirm normality of our data distribution. One-way analysis of variance (ANOVA) tests (with Tukey’s multiple comparisons) were run to find any significant differences in the mean numbers of immune cell subpopulations at each time-point collection. Immune cell counts in patients who suffered symptomatic VSP were compared to those whom did not. Cytokine concentrations from ELISA essays were analyzed in a similar fashion. Due to small sample size, we did not perform comprehensive uni- or multi-variable logistic regression analyses correlating immune cell changes with severity of VSP. *P*-values < 0.05 were considered statistically significant. All statistical analyses were performed with GraphPad Prism v8 (San Diego, CA, USA).

## Results

### Patient and aneurysm characteristics

Twenty patients were screened. Six patients with non-aneurysmal SAH and one patient with human immunodeficiency virus were excluded. Thirteen patients/aneurysms were included over a period of seven months (Table 1). Five (38.5%) patients developed VSP: 2 cases mild and 3 cases moderate; no severe VSP cases were encountered. Mean time from aSAH to VSP was 163.2 ± 72.8 h (6.8 days). One patient died secondary to sudden cardiac arrhythmia.

**Table 1:** Patients’ characteristics.

N	Age/sex	Size	Location	mFS	WFNS	Treatment	VSP	Day of VSP
1	65/M	3	Pericallosal	1	1	Coiling	No	–
2	66/F	6	BA tip	4	5	Coiling + WEB	No	–
3^†^	88/F	5.4	PCOM*	4	5	Coiling	No	–
4	67/F	5	ACOM*	4	5	Coiling	Moderate	4
5	33/F	7.4	PCOM*	1	1	Coiling	No	–
6	58/F	6.5	BA tip*	4	2	SAC	No	–
7	50/M	3	ACOM	1	1	Coiling	No	–
8	50/M	5	MCA*	4	4	Coiling	Moderate	9
9	53/F	3.2	ACOM	4	4	Coiling	Mild	9
10	45/F	5	ACOM	3	2	Coiling	No	–
11	39/M	3.8	PCOM	1	1	Coiling + FD	Moderate	9
12	53/F	3.5	BA tip*	4	4	BAC	No^‡^	–
13	65/M	6.1	MCA	4	5	Coiling	Mild	3

*ACOM* anterior communicating artery; *BA* basilar artery; *BAC* balloon-assisted coiling; *FD* flow diversion; *M* male; *MCA* middle cerebral artery; *mFS* modified Fisher Scale; *PCOM* posterior communicating artery; *SAC* stent-assisted coiling; *VSP* cerebral vasospasm; *WFNS* World Federation of Neurological Surgeons grading scale.

*Aneurysm with bleb/daughter sac.

^†^Deceased.

^‡^Patient experienced *clinically silent radiographic* VSP.

### Immune cell kinetics in aneurysmal subarachnoid hemorrhage

**Figure 1:**
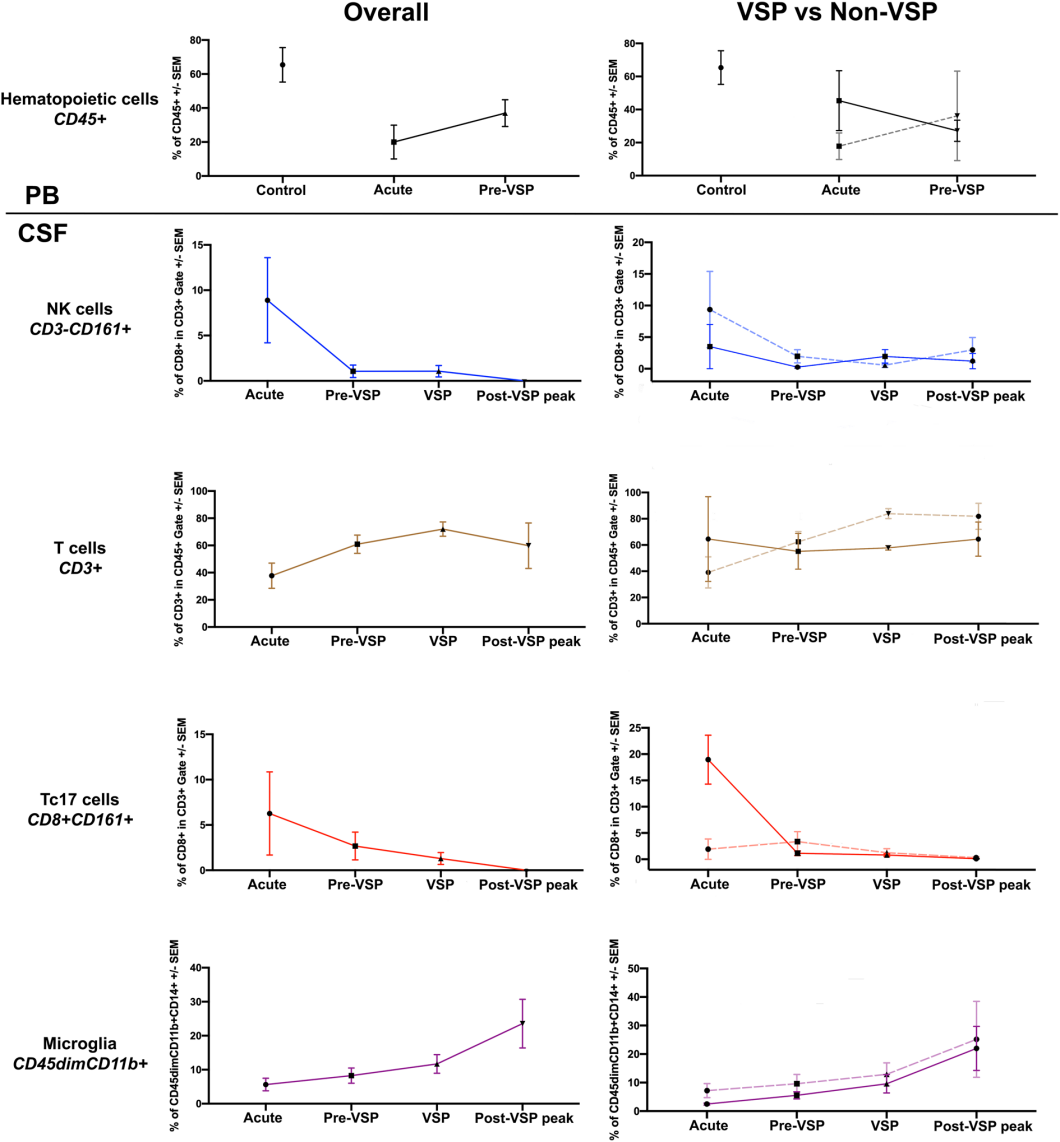
Immune cell kinetics in aneurysmal subarachnoid hemorrhage. For the right column, patients with vasospasm (VSP) are depicted in continuous lines, whereas non-VSP patients are shown in dashed lines. *PB* peripheral blood; *CSF* cerebrospinal fluid.

**Figure 2:**
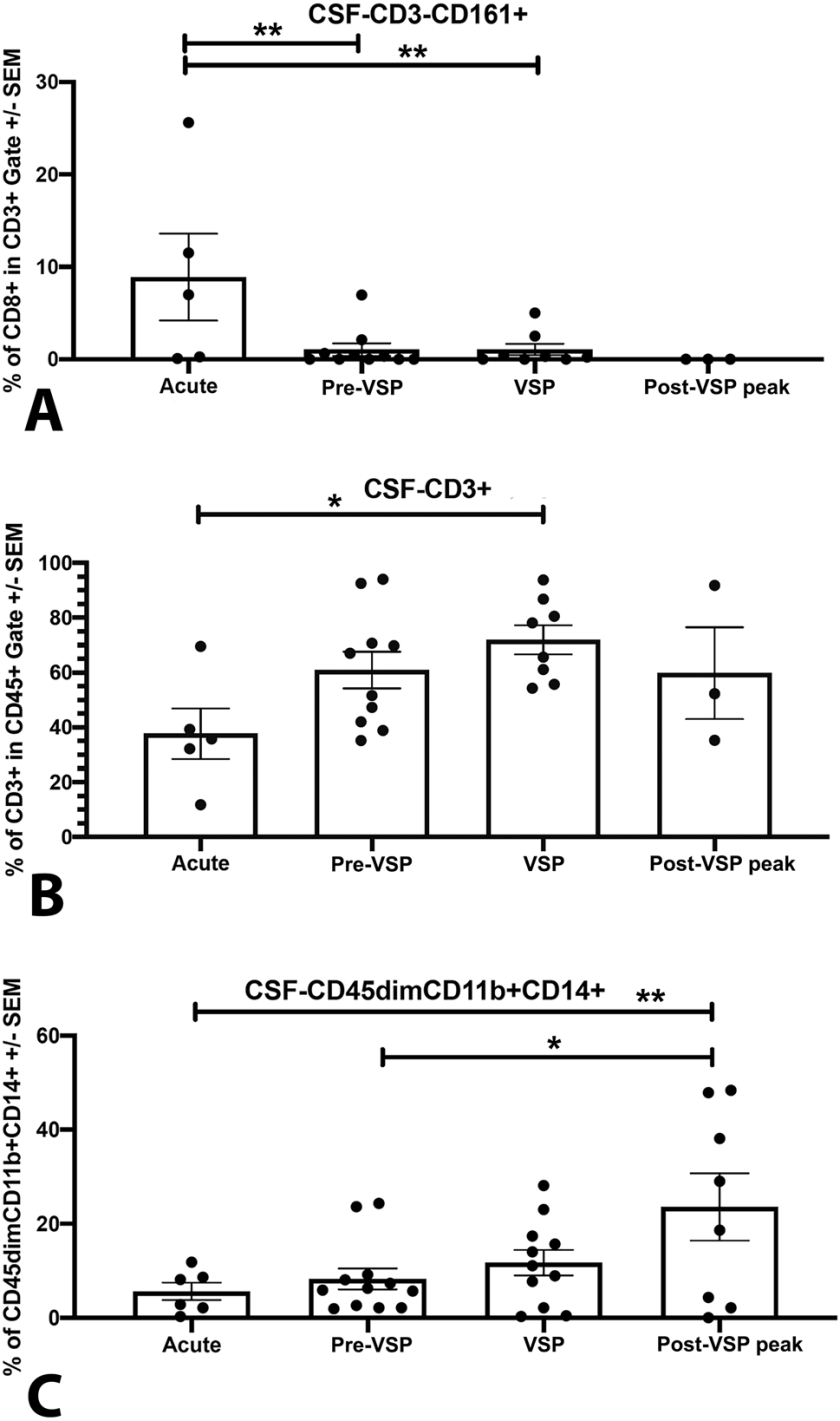
CSF cellularity for (**A**) CD3-CD161 + NK cells, (**B**) CD3 + T-cells, and (**C**) CD45dimCD11b + microglia.

Brown-Forsythe test demonstrated no significant differences in standard deviations of immune cell counts at each time-point among patients with and without VSP (*P* > 0.05). ANOVA tests showed that during the acute phase (days 0–1), aSAH patients had significantly decreased numbers of lymphohematopoietic CD45+ cells when compared to healthy controls (*P* = 0.0101; Table 2, Supplemental Fig. 3A); this “acute immunosuppression” was particularly significant in patients without VSP (*P* = 0.0373; Supplemental Fig. 3B). CSF analysis demonstrated elevated counts of natural killer (NK) cells (CD3-CD161 +) during the acute phase, followed by a gradual reduction (*P* = 0.0419; Figs. 1, 2A) of cell numbers. During VSP, the number of CSF T-cells (CD3 +) was significantly higher when compared to the acute phase (*P* = 0.0333; Figs. 1, 2B). The number of CSF microglia (CD45dimCD11b +) cells progressively increased over time after aSAH (*P* = 0.0326; Figs. 1, 2C).

**Table 2:** Evolution of immune cells and cytokine mediators in human CSF after aSAH.

Immune cell/cytokine	Acute (days 0–1)	Pre-VSP (days 2–4)	VSP (days 5–9)	Post-VSP peak (days 10 +)
CD45 + Hematopoietic cells	↓↓↓	↑	↑↑	↑↑↑
CD3-CD161 + NK cells	↑↑	↓	↓↓	↓↓↓
CD3 + T-cells	↑	↑↑	↑↑↑	↓
**CD8 + CD161 + Tc17 cells**	**↑↑↑↑**	**↓**	**↓↓**	**↓↓↓**
**CD45dimCD11b + Microglia**	**↑**	**↑↑**	**↑↑↑**	**↑↑↑↑**
VEGF	↑↑	↓	↓↓	↑
MMP-9	↑↑	↓	↓↓	↑

Highlighted in bold are the immune cell subpopulation with most significant count changes.

*VSP* cerebral vasospasm; *MMP-9* matrix metalloproteinase-9; *NK* natural killer; *VEGF* vascular endothelial growth factor.

**Figure 3:**
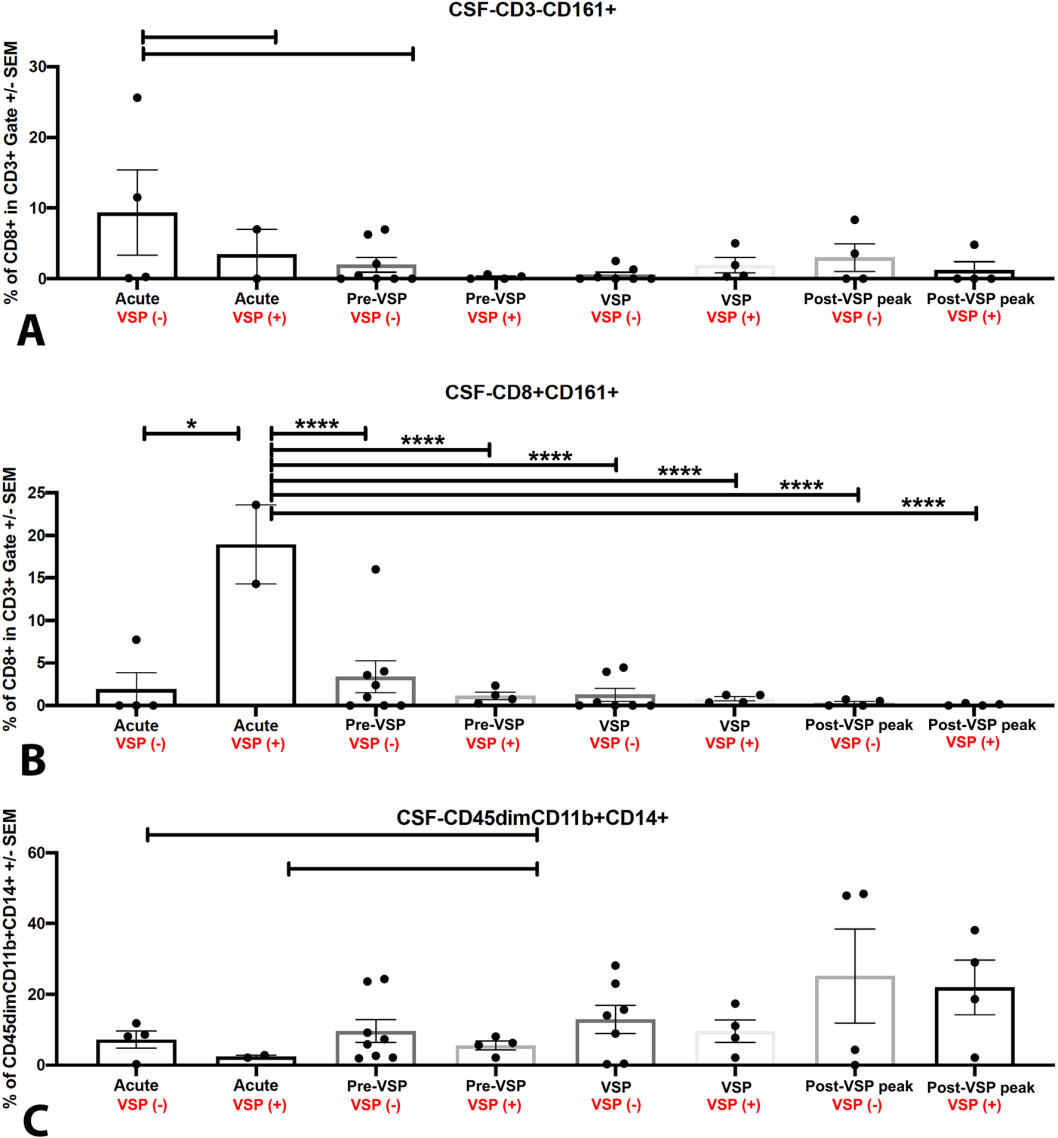
Comparison of CSF cellularity among patients with and without VSP for (**A**) CD3-CD161 + NK cells, (**B**) CD8+ CD161+ Tc17 cells, and (**C**) CD45dimCD11b + microglia.

CSF cellularity was compared among patients with and without VSP. Patients with VSP had lower numbers of CSF NK cells (CD3-CD161 +) in the acute phase compared to patients without VSP (*P* > 0.05; Figs. 1, 3A). The analysis of T cell subpopulations demonstrated significantly increased numbers of CD8+CD161+ cells during the acute phase in patients who developed VSP compared with those who did not (*P* < 0.0001; Figs. 1, 3B). In patients with VSP, the number of CSF microglial cells was low in the acute and pre-VSP phases, with a progressive increase during the post-VSP peak (*P* > 0.05; Figs. 1, 3C).

### Inflammatory mediators and cytokine kinetics in aneurysmal subarachnoid hemorrhage

**Figure 4:**
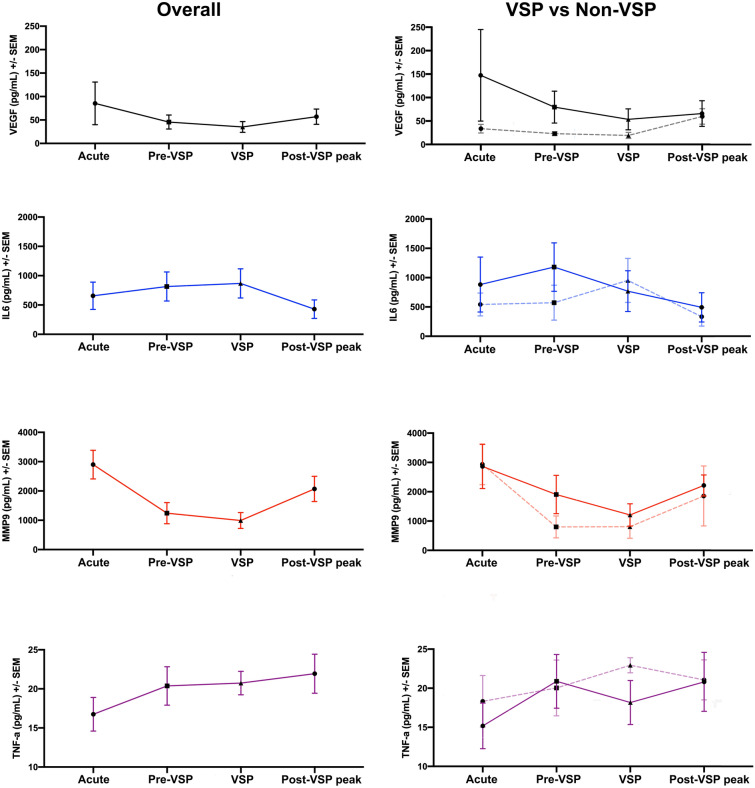
Inflammatory mediators and cytokine kinetics in aneurysmal subarachnoid hemorrhage. For the right column, patients with vasospasm (VSP) are depicted in bold lines, whereas non-VSP patients are shown in dashed lines.

ELISA demonstrated higher levels of VEGF in the acute phase in aSAH patients compared with healthy controls (*P* > 0.05; Fig. 4 and Supplemental Fig. 4A). At all time-points, VEGF levels showed a higher trend in CSF from patients with VSP compared to those without VSP (*P* > 0.05; Supplemental Fig. 4B). Lower levels of MMP-9 were found in CSF from aSAH patients at pre-VSP and VSP time-points compared with the acute phase (*P* = 0.0248 and 0.0089, respectively; Fig. 4 and Supplemental Fig. 3C). There were no significant differences in the expression of IL-6 or TNF-α (Fig. 4 and Supplemental Fig. 5).

### Confocal immunofluorescence imaging of unruptured intracranial aneurysms

**Figure 5:**
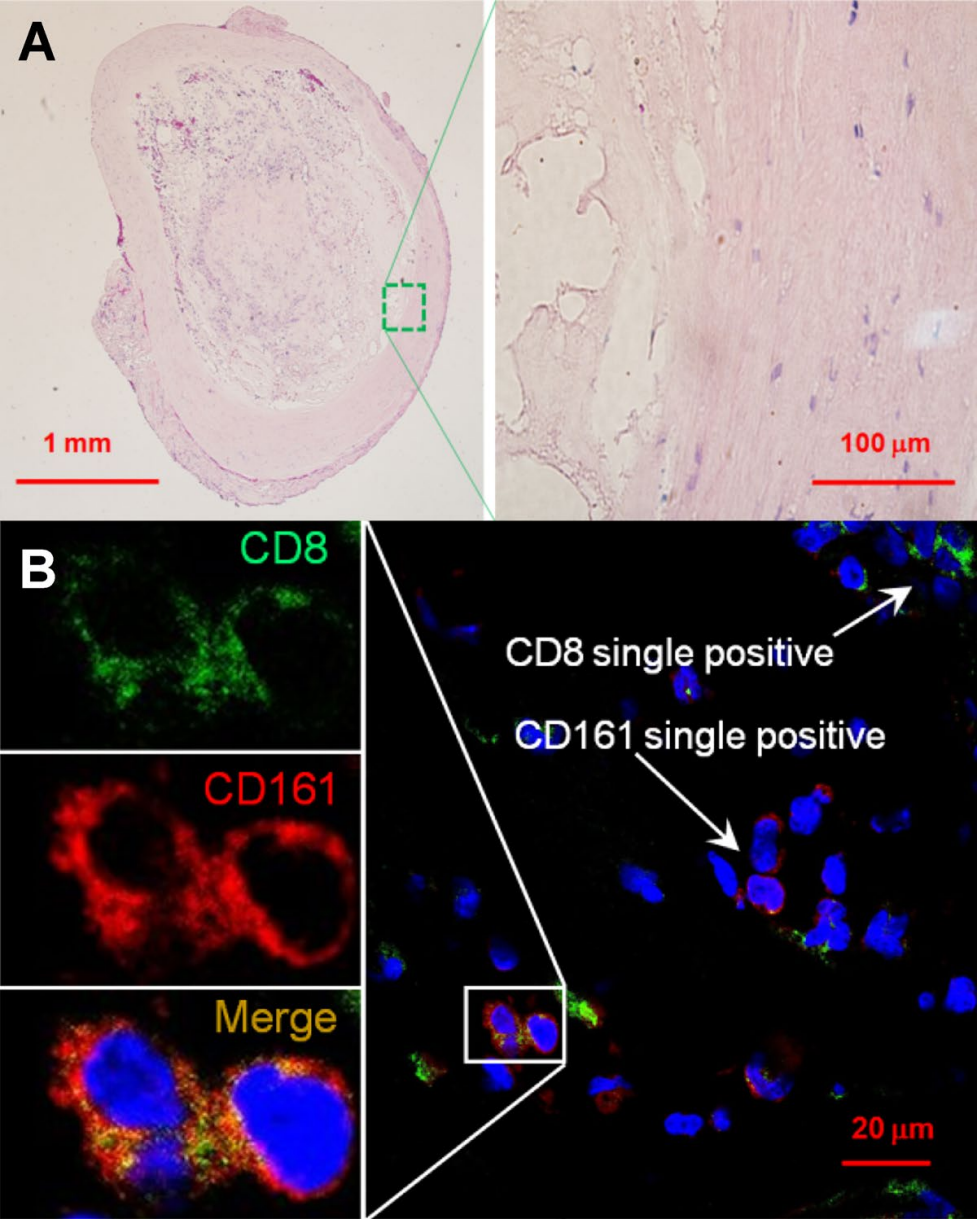
Confocal immunofluorescent analysis of unruptured intracranial aneurysms. (**A**) Hematoxylin and eosin staining of a specimen’s section. (**B**) Immunofluorescence imaging of anti-CD8 (green) and anti-CD161 (red) staining. Nuclei were stained by DAPI (blue). The area with the co-localization of CD8+ and CD161+ was zoomed-in to show CD8 and CD161 expression in separated and merged confocal images. Scales (in μm) are denoted by the bars.

Since the preliminary flow cytometry data demonstrated predominance of *CD8*+*CD161*+ *cells* in the CSF of patients with VSP, we performed immunostaining of histological samples previously collected from two unruptured aneurysms with the following primary antibodies: anti-human CD8 (mouse monoclonal, Cat # ab17147, Abcam, Cambridge, MA) and anti-human CD161 (rabbit polyclonal, Cat # ab197979, Abcam, Cambridge, MA). Tissue sections were washed twice in phosphate-buffered saline and incubated simultaneously with two secondary antibodies for immunofluorescence: goat anti-mouse Alexa Fluor 488 (Cat # 21042) and goat anti–rabbit Alexa Fluor 568 (Cat # 11011, ThermoFisher Scientific, Waltham, MA). Samples were washed three times with PBS and mounted (VECTASHIELD Antifade Mounting Medium with DAPI, Cat # H-1200, Vector Laboratories, Burlingame, CA). Confocal immunostaining indicated the presence of CD8+CD161+ cells in the arterial wall of both unruptured aneurysms (Fig. 5).

## Discussion

**Figure 6:**
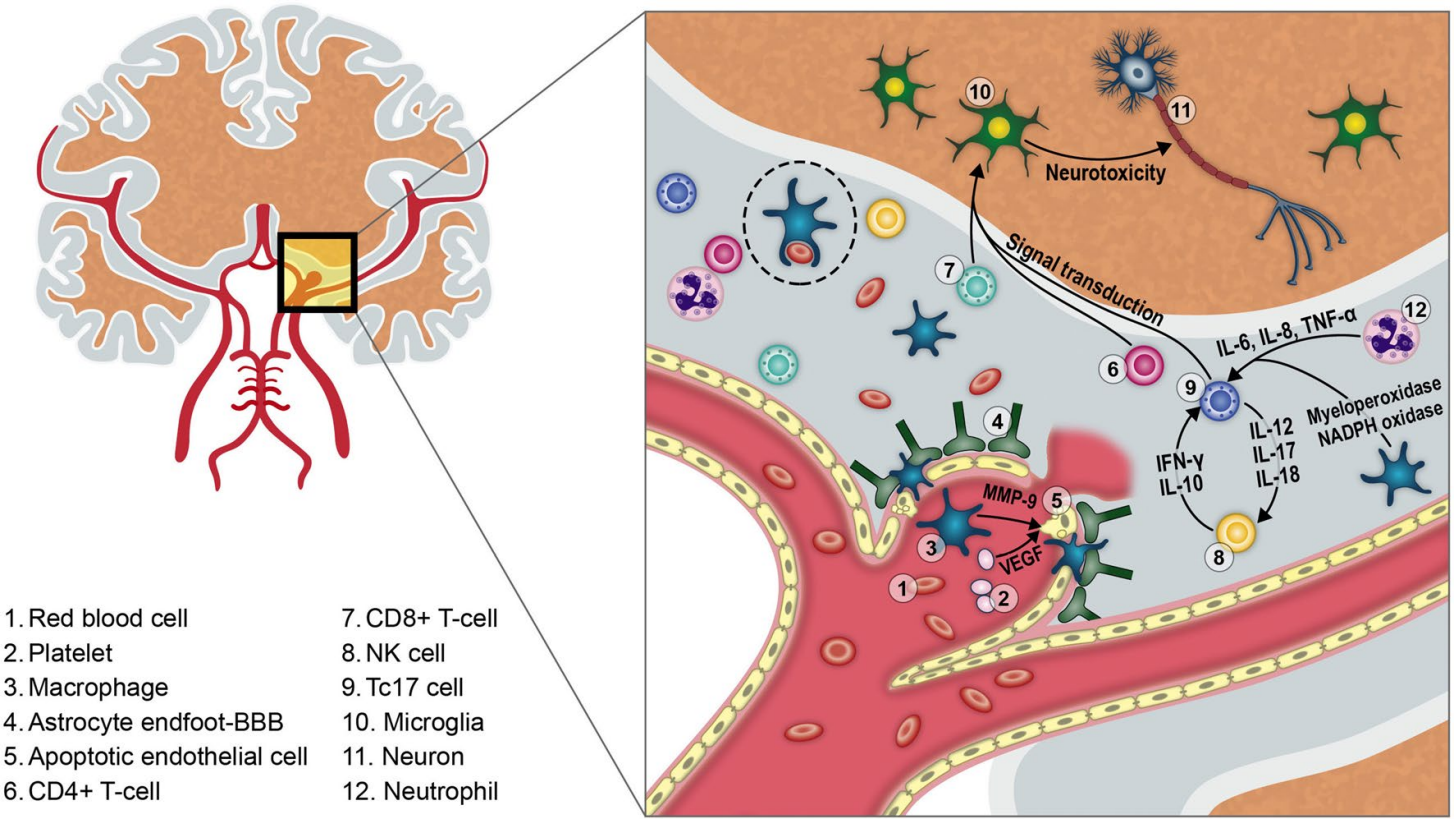
The immune response following aSAH induces inflammation of the aneurysmal wall, apoptosis of endothelial cells and degradation of tight junctions. This increases the permeability of the BBB and allows active extravasation of immune cells into the subarachnoid space. Once in the CSF, cells of the innate immune system (mainly neutrophils and macrophages) phagocytose red blood cells/debris (dotted ellipse) and secrete multiple cytokines that stimulate CD4+ and CD8+ T-cells from the adaptive immunity. This might perpetuate the intrathecal inflammatory response by production of IL-17 from CD8+CD161+ cells. Finally, microglial cells in the brain parenchyma become activated and, instead of conferring protection from further damage, induce secondary neurotoxicity.

This preliminary study prospectively assessed the immunological response after aSAH in human CSF. Our results suggest that both innate and adaptive immune responses play pivotal roles after aSAH. The adaptive immune response seems to be primarily mediated by *CD8*+*CD161*+ *cells*, which significantly correlated with the occurrence of VSP. Confocal immunostaining suggested the presence of this subpopulation of cytotoxic T cells in the aneurysmal wall. Microglia proliferation seems to occur in a delayed fashion around 6 + days after aSAH (Fig. 6).

### Acute immunosuppression after aneurysmal subarachnoid hemorrhage

The concentration of CD45+ cells in PB after aSAH was lower in aSAH patients compared with healthy controls. This finding is compatible with the *acute systemic immunosuppressive response* after aneurysmal rupture previously suggested by other studies. Sarrafzadeh et al. described T cell-lymphopenia and a decreased monocyte human leukocyte antigen-DR expression on PB of patients after aSAH (i.e. “aSAH-induced acute immunodepression state”)^23^. However, our results also suggest that the early decrease in CD45+ cells is particularly significant in patients without VSP, whereas patients with VSP show *less immunosuppression* during the acute phase. We hypothesize that the lack of systemic immunosuppression after aSAH may increase the risk of developing VSP due to a *sustained pro-inflammatory response*. This systemic effect may be magnified in the central nervous system after disruption of the BBB.

### Innate immune system response after aneurysmal rupture

Innate immune cells enter the subarachnoid space after aSAH. This process occurs via increased expression of cellular adhesion molecules (E-selectin, VCAM-1, ICAM-1 and HMGB-1) and has been correlated with the occurrence of VSP^24–27^. Once in the subarachnoid space, activated cells secrete multiple cytokines such as IL-1β, IL-6, TNF-α, LFA-1, leukotrienes, arachidonic acid, von Willebrand factor, complement, MMP-9 and VEGF^28–31^. These chemo-attractants may trigger and/or worsen VSP^32^. We found a peak level of MMP-9 during the acute phase compared with the pre-VSP and VSP phases. MMP-9 is a key neurovascular protease that can induce BBB damage and cause edema, hemorrhage and neuronal death^33^. Egashira et al. found less white matter injury in MMP-9 knock-out mice compared with wild-type mice 8 days after induction of SAH^34^. Using quantitative reverse PCR in PB samples of 43 SAH patients and 23 healthy controls, Wang et al. detected increased levels of MMP-9 in SAH patients who developed VSP^31^.

Additionally, patients with VSP showed a trend to express higher levels of VEGF compared with those without VSP. VEGF is a key factor involved in revascularization, endothelial cell migration and proliferation. Several studies have suggested that increased VEGF levels reduce the integrity of the BBB by weakening tight junctions via the VEGFR-2 located on endothelial cells^35^. Liu et al. found that anti-VEGF antibodies decreased BBB permeability and ameliorated brain edema/injury after SAH induced in mice^36,37^. In conjunction with our pilot data, this evidence supports the hypothesis that active BBB injury mediated by MMP-9 and VEGF occurs early in aSAH, and may contribute to VSP and DCI by promoting neuroinflammation.

### Adaptive immune system response after aneurysmal rupture

The role of the adaptive immunity after aSAH has not been thoroughly characterized. In 1993, Mathiesen et al. reported moderately increased levels of IL-2 receptor in patients after aSAH. No systemic changes in IL2 were documented, suggesting a selective intrathecal synthesis^38^. IL-2 receptor is expressed on the surface of lymphocytes, and its intracellular signal transduction has a crucial role in the development of memory T cells and terminal differentiation of effector T cells^39^. Although this study was the first to suggest activation of adaptive immunity in aSAH, a limitation was the inclusion of subjects with traumatic SAH and non-aneurysmal spontaneous SAH (n = 4/12). In 1996, the same group analyzed lymphocyte subpopulations from 10 CSF samples of aSAH patients and found an increase of CD3+ cells in 2 patients, CD4+ cells in 1 patient, CD8+ cells in 3 patients, and CD19+ cells in 3 patients^18^. The development of VSP was not assessed.

Recently, Moraes et al. analyzed CSF and PB samples from 12 aSAH patients and PB samples from 28 healthy controls. In aSAH patients, CD4+ and CD8+ T cells had decreased expression of CD3/CD28 and higher levels of CD69 in CSF compared with PB samples. This suggests a chronic activation of adaptive immune cells in the central nervous system^19^. The authors analyzed two CSF samples collected at different time-points after aSAH (1–3 and 4–6 days) in five patients: no differences in the number or activation status of immune cells were found.

Our study determined increased numbers of CSF T cells (CD3 +) during VSP compared with the acute phase. Specifically, *CD8*+*CD161*+ *cells* increased significantly in the CSF of patients who developed VSP compared to those who did not have clinical VSP. CD8 is a surface marker of cytotoxic T cells, whereas CD161 (a C-type lectin member of the human NKR-P1 family) is mainly expressed in NK cells and T cells. CD8+ T cells expressing high levels of CD161 synthesize a pattern of molecules similar to the type-17 phenotype^40^. Thus, these cells are also known as “*Tc17*” cells and have been shown to secrete IL-17 induced by activation of the transcription factor retinoic acid-related orphan receptor C (RORC) pathway^41^.

The main function of IL-17 is to increase the local production of chemokines and recruit monocytes to the site of inflammation^42^. IL-17 produced by *CD8*+*CD161*+ *cells* has been shown to contribute to the inflammatory response in experimental murine models of multiple sclerosis and autoimmune encephalitis^43,44^. However, the role of IL-17 in aSAH and VSP remains poorly understood. Our group has previously demonstrated increased concentrations of IL-17 in blood samples from the lumen of human cerebral aneurysms compared with samples from the femoral artery^45^. Chaudhry et al. also showed systemic upregulation of the IL-23/IL-17 inflammatory axis in human PB samples after aSAH^46^. Using an estrogen-deficient mice model, Hoh et al. recently demonstrated that IL-17A-blockade exerts a protective effect against aneurysm formation and rupture by increased E-cadherin expression^58^.

Previous evidence has shown active infiltration of the aneurysmal wall by cells of the innate immune system (e.g. macrophages, neutrophils)^47^. However, our flow cytometry analysis of post-hemorrhagic CSF from patients with VSP suggested activation of *CD8*+*CD161*+ *“Tc17”* lymphocytes. Thus, we aimed to identify the potential source of these cells by analyzing tissue of brain aneurysms. We demonstrated the presence of Tc17 cells in the wall of two unruptured aneurysms with confocal imaging. This finding suggests that: (1) the aneurysmal wall may be the source of Tc17 cells seen in the subarachnoid space after aneurysm rupture; and (2) Tc17 cells may be an important source of IL-17 and pro-inflammatory cytokines both in the vascular wall of unruptured aneurysms and CSF after aSAH. Expression of CD161 on the surface of CD8+ T cells stimulates high expression of transcription factors that induce differentiation of more Tc17 cells nearby^48^. Further research is needed to establish the role of CD8+CD161+ cells as a potential immunomodulatory target to prevent VSP after aSAH.

### Microglia and secondary brain damage

VSP is not directly correlated with the development of DCI^49^. Several randomized controlled trials failed to demonstrate long-term improvement of clinical outcomes in patients with aSAH after administration of different anti-vasospasm medications^21,50–54^. DCI may be the result of a neuroinflammatory response mediated by microglial cells after aSAH. Microglia are the cellular mediators of inflammation in the central nervous system; they exist in a quiescent state and become activated following exposure to an insult or infection^55^. Hanafy et al. demonstrated in mice that neuronal apoptosis and VSP on day 7 after induction of SAH was mainly microglial-dependent, and became microglial-independent at latter stages^56^. Similarly, Schneider et al. showed a wave of Iba-1-positive cells spreading within mice brain tissue between days 4–28 after experimental SAH. The wave of immune cells was chronologically correlated with neuronal cell death^13^.

In our study, the number of CSF microglia was low immediately after aSAH. However, this cell population progressively increased during VSP and post-VSP peak periods. In chronic neurodegenerative processes such as Alzheimer’s/Parkinson’s disease and multiple sclerosis, it has been demonstrated that microglial cells remain activated for long periods of time and continue to release inflammatory mediators indefinitely^57^. These cells may have a role in the development of post-hemorrhagic VSP and DCI. The role of microglia in the secondary brain damage seen months to years after aSAH remains undetermined.

### Limitations

The main limitation of this study is the small number of patients. However, we collected CSF samples at 4 different time-points and a PB sample at one point per study subject. The immunological profile changed over time and reached statistical significance despite the limited number of patients. The results of this pilot-exploratory study will need to be confirmed in a larger cohort.

Most patients had severe aSAH (61% = mFS 4), therefore the sample is biased towards sicker patients with larger hemorrhages. Additionally, one subject in the no-VSP group experienced clinically silent radiographic vasospasm (patient #12, Table 1). Whether these vascular changes are immune/inflammatorily mediated or not remains unknown^2^. Moreover, vasospasm is part of the spectrum of processes of DCI. Other events such as cortical spreading ischemia, microthrombosis and constriction of the microcirculation are difficult to quantify and were not assessed in this study.

Two patients underwent SAC and flow diversion for aneurysm treatment and required dual antiplatelet therapy. Aspirin may decrease the inflammatory response of these patients and may have altered the expansion of immune cell populations”^58,59^.

We did not quantify CSF IL-17 levels due to lack of enough sample volumes. Further investigation to better characterize the role of activated *CD8*+*CD161*+ cells in aSAH would require comparison of IL-17 production profiles using intracellular cytokine staining or gene expression patterns in RNA sequencing. The other limitation is the sampling of PB at only the first time-point of the study; future studies should sample PB longitudinally to compare immunological systemic and CSF responses.

## Conclusion

Innate and adaptive immune cells play a pivotal role after aSAH. This preliminary study demonstrated that *CD8*+*CD161*+ lymphocytes increase over time in the CSF of patients after aSAH. The number of cells is higher during VSP. Moreover, these cells were identified in the wall of two unruptured intracranial aneurysms. Microglia activation occurs 6 + days after aSAH.

## Supplementary information

Supplementary file1 (PDF 1530 kb)

## Data Availability

Data will be made available upon request from the corresponding author.
