# Supplementary material for: Preliminary results in the analysis of the immune response after aneurysmal subarachnoid hemorrhage
Source: Sci Rep. 2020 Jul 16;10:11809. doi: 10.1038/s41598-020-68861-y (PMC7367262; doi:10.1038/s41598-020-68861-y)

Preliminary Results In The Analysis Of The Immune Response After Aneurysmal Subarachnoid Hemorrhage

Jorge A. Roa MD[1,2], Deepon Sarkar BS[1], Mario Zanaty MD[2], Daizo Ishii MD[2], Yongjun Lu PhD[2], Nitin J. Karandikar MD PhD[3], David M. Hasan MD[2], Sterling B. Ortega PhD[3], Edgar A. Samaniego MD, MS[1, 2, 4]

1. *Department of Neurology, University of Iowa Hospitals and Clinics, Iowa City, Iowa, USA.*
2. *Department of Neurosurgery, University of Iowa Hospitals and Clinics, Iowa City, Iowa, USA.*
3. *Department of Pathology, University of Iowa Hospitals and Clinics, Iowa City, Iowa, USA.*
4. *Department of Radiology, University of Iowa Hospitals and Clinics, Iowa City, Iowa, USA.*

Supplemental Figure 1. Timeline for CSF and PB sample collection.

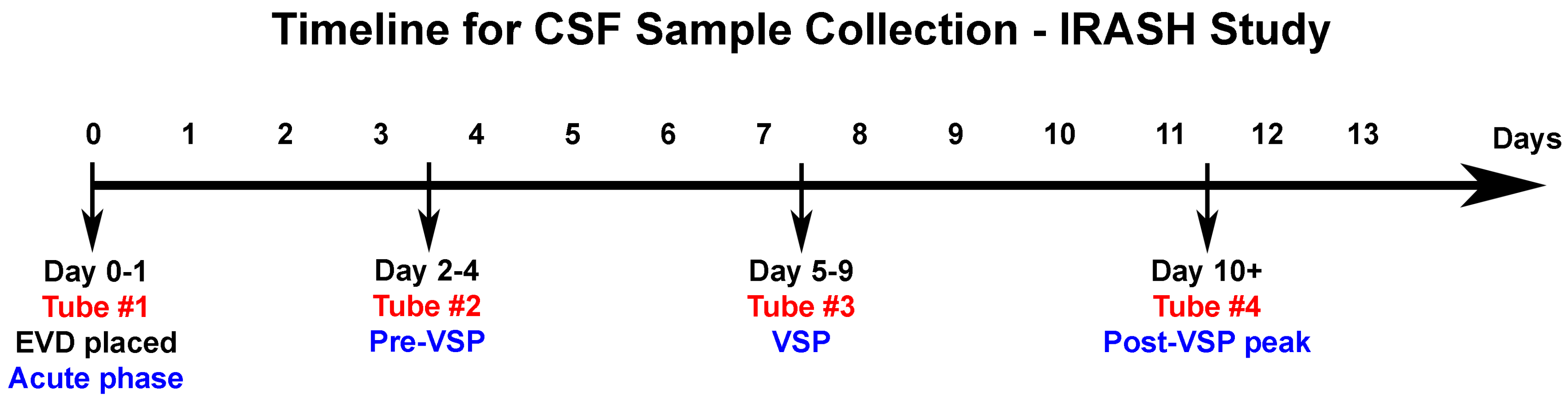

Supplemental Figure 2. Gating strategy used during flow cytometry.

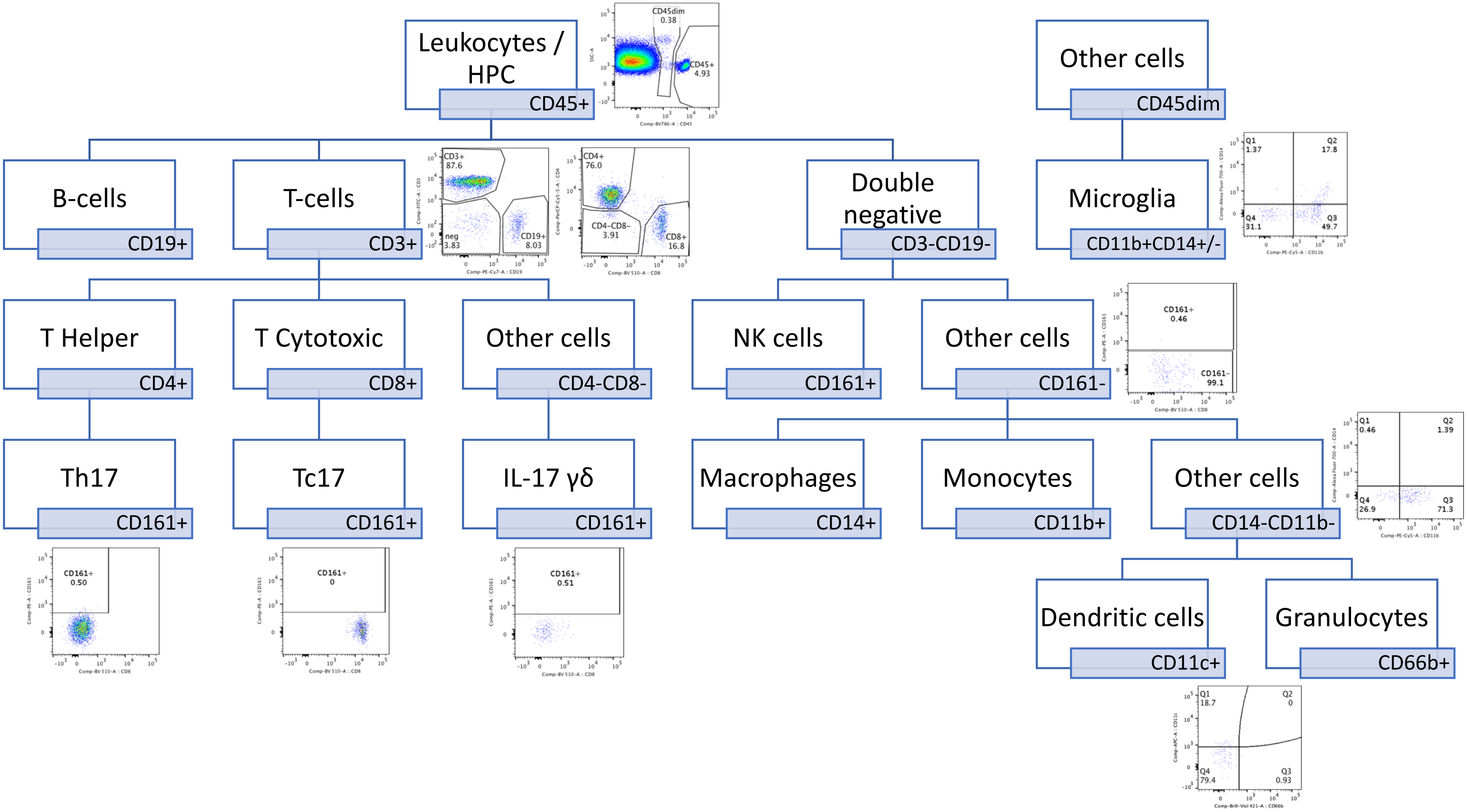

Supplemental Figure 3. PB CD45+ cellularity (A) among all patients and (B) categorized by presence of VSP.

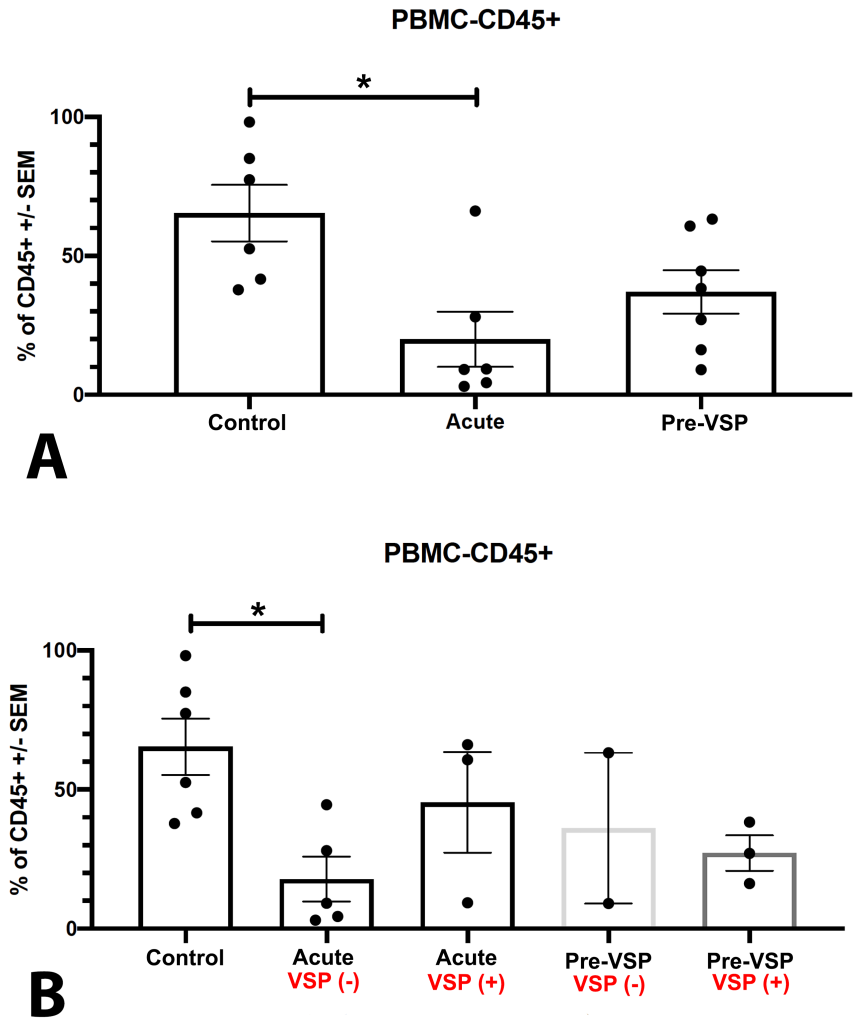

Supplemental Figure 4. ELISA results for expression of (A) VEGF among all patients, (B) VEGF categorized by presence of cVSP, and (C) MMP-9 among all patients.

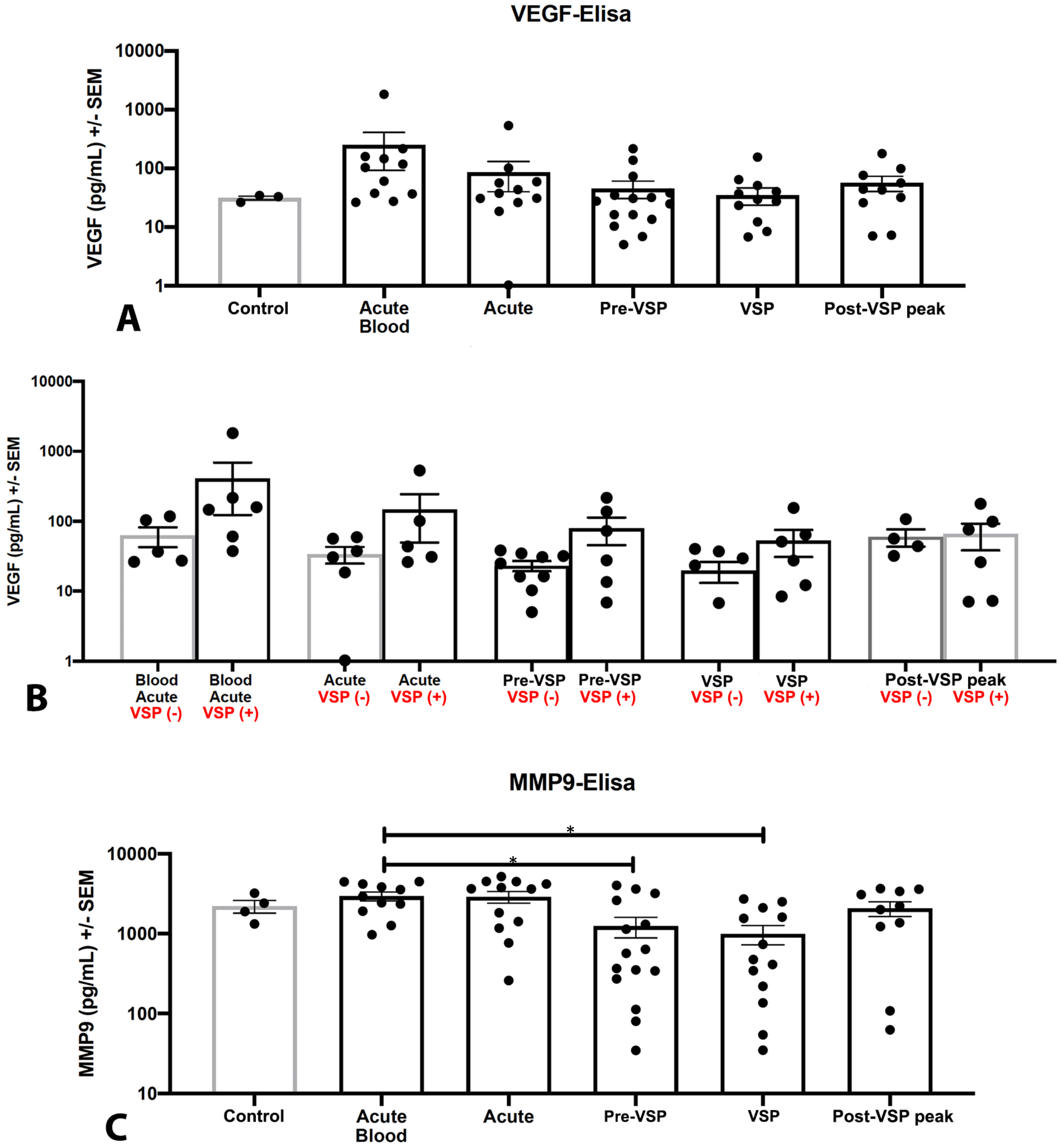

Supplemental Figure 5. ELISA results for expression of (A) IL-6 among all patients, (B) IL-6 categorized by presence of cVSP, and (C) TNF-α among all patients.

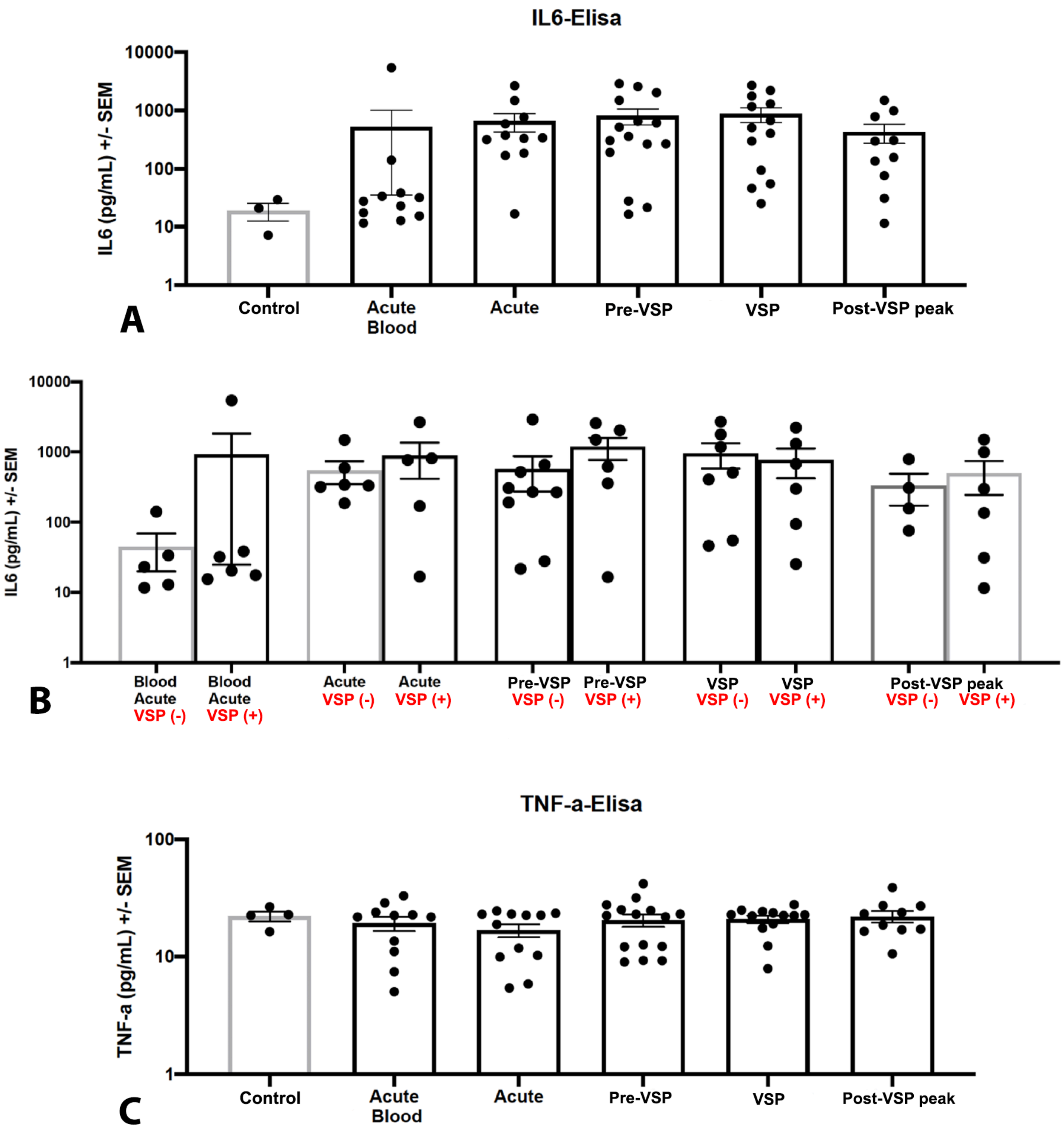

Supplement: Supplementary file 1 — Supplementary file1 (PDF 1530 kb) [file 41598_2020_68861_MOESM1_ESM.pdf]
